# Effects of Self‐Perceptions of Cognitive Decline on Cognitive Assessment Outcomes in the Women’s Alzheimer’s Movement Prevention Center at Cleveland Clinic

**DOI:** 10.1002/alz.092611

**Published:** 2025-01-09

**Authors:** Nikki L Kaplan, Emily Post, Taylor F Levine, Jessica ZK Caldwell

**Affiliations:** ^1^ Cleveland Clinic Lou Ruvo Center for Brain Health, Las Vegas, NV USA

## Abstract

**Background:**

The Women’s Alzheimer’s Movement Prevention Center (WAMPC) at Cleveland Clinic evaluates women’s risk factors for developing Alzheimer’s disease and provides personalized recommendations for reducing risk. Previous research has established that up to 40% of Alzheimer’s disease (AD) cases might be prevented when modifiable risk factors are addressed. To better understand individual risk profiles, a wealth of information is collected, including self‐reported health conditions (both personal and familial), lifestyle factors (e.g. alcohol and tobacco use, exercise, social support), a brief cognitive test battery, and blood tests. WAMPC takes a holistic approach in helping women combat modifiable risks for AD.

**Method:**

207 women from the WAMPC who consented to have their clinical data used for research purposes were assessed (mean age = 53.39, SD = 9.53). Requirements for participation include normal cognition and family history of Alzheimer’s disease. Patients completed the Mini Mental State Exam (MMSE), selected tests from the NIH Toolbox (Oral‐Symbol Digit, Processing Speed, Picture Vocabulary, Card Sort, and Flanker), Controlled Oral Word Association Test‐FAS (COWAT: FAS, Animal Naming), and the Trail Making Test Part B. Patients also completed the Subjective Cognitive Decline Questionnaire (SCDQ), a self‐report assessment of perceived cognitive changes. We transformed SCDQ total scores into cohort‐based z‐scores to create three SCDQ groups (“fewer cognitive concerns” = bottom third, “average cognitive concerns” = middle third, and “above average cognitive concerns” = top third. We evaluated the relationship between performance on cognitive tests and women’s perceived cognitive decline on SCDQ. We ran 9 individual ANCOVAs, one for each cognitive test, using education and age as covariates.

**Result:**

Of the tests evaluated, the SCDQ significantly predicted scores on Animal Naming (F(2,190) = 3.602, p = .029) and NIH Toolbox Card Sorting (F(2,190) = 3.597, p = .029). No other tests demonstrated significant relationships with self‐perceptions of cognitive decline per the SCDQ.

**Conclusion:**

Perceived cognitive decline in daily life predicted concurrent poorer performance on selected tests of language and executive function, in women who are cognitively normal, yet at increased risk for AD based on family history. Further work is needed to understand whether subjective cognitive concerns may represent risk for cognitive decline in this cohort of women with family history of AD.

